# Eravacycline as Salvage Therapy for Severe Intra-Abdominal Infections Caused by Multidrug-Resistant *Acinetobacter baumannii*: A Case Series

**DOI:** 10.3390/antibiotics15010093

**Published:** 2026-01-16

**Authors:** Marcello Trizzino, Giulio D’Agati, Luca Pipitò, Claudia Conti, Rossella Petrantoni, Raffaella Rubino, Antonio Anastasia, Sofia Urso, Irene Ganci, Maria Cappello, Antonio Cascio

**Affiliations:** 1 Infectious and Tropical Diseases Unit, Sicilian Regional Reference Center for the Fight Against AIDS, AOU Policlinico “P. Giaccone”, 90133 Palermo, Italy; marcello.trizzino@policlinico.pa.it (M.T.); giulio.dagati@community.unipa.it (G.D.); claudia.conti@community.unipa.it (C.C.); rossella.petrantoni@policlinico.pa.it (R.P.); raffaella.rubino@policlinico.pa.it (R.R.); antonio.anastasia@policlinico.pa.it (A.A.); sofia.urso@community.unipa.it (S.U.); irene.ganci@community.unipa.it (I.G.); 2 Department of Health Promotion, Mother and Child Care, Internal Medicine and Medical Specialties “G D’Alessandro”, University of Palermo, 90133 Palermo, Italy; 3 Gastroenterology & Hepatology, AOUP “P. Giaccone”, Università di Palermo, 90127 Palermo, Italy; maria.cappello@policlinico.pa.it

**Keywords:** eravacycline, *Acinetobacter baumannii*, multidrug-resistant, intra-abdominal infection, carbapenem-resistant, case report

## Abstract

**Background/Objectives**: Infections due to multidrug-resistant (MDR) *Acinetobacter baumannii* represent a critical challenge in modern healthcare, with limited therapeutic options. Eravacycline, a novel fluorocycline antibiotic, demonstrates promising in vitro activity, but real-world clinical data for complex intra-abdominal infections (IAIs) are scarce. We present two cases of severe IAI caused by carbapenem-resistant *A. baumannii* (CRAB) successfully treated with eravacycline. **Methods**: We describe the clinical course, microbiological findings, and outcomes of two critically ill patients. Case 1 was a 75-year-old male with biliary peritonitis following an endoscopic procedure. Case 2 was a 64-year-old male with infected pancreatic walled-off necrosis. Both patients had cultures positive for CRAB and failed multiple prior antibiotic regimens. **Results**: In both cases, the initiation of intravenous eravacycline led to significant clinical improvement, including resolution of septic shock and defervescence. A marked reduction in inflammatory markers (C-reactive protein and procalcitonin) was observed, alongside microbiological clearance of CRAB. Eravacycline was well tolerated, with no significant adverse events. **Conclusions**: These case reports suggest that eravacycline can be an effective and safe salvage therapy for complex IAIs caused by CRAB, even in scenarios of partial source control. It represents a valuable addition to the antimicrobial armamentarium for managing infections caused by these extensively drug-resistant organisms.

## 1. Introduction

Multidrug-resistant (MDR) *Acinetobacter baumannii* is a leading cause of healthcare-associated infections, particularly in intensive care units, posing a severe threat due to its association with high mortality and substantial healthcare costs [1,2]. The organism’s remarkable ability to acquire resistance mechanisms, including the production of carbapenem-hydrolyzing class D β-lactamases (OXA-type) and metallo-β-lactamases (MBLs), often results in strains resistant to nearly all first-line antibiotics, thereby classifying them as difficult-to-treat resistant (DTR) pathogens [3]. Complicated intra-abdominal infections (cIAIs) caused by such strains represent a therapeutic dilemma, as the efficacy of conventional regimens is profoundly compromised.

The current therapeutic armamentarium for carbapenem-resistant *A. baumannii* (CRAB) infections is limited and often relies on salvage agents like colistin and tigecycline [4,5]. More recently, cefiderocol and the β-lactam/β-lactamase inhibitor combination sulbactam–durlobactam have emerged as important additional options for CRAB infections, with sulbactam–durlobactam now recommended as a preferred regimen in combination with a carbapenem in the latest IDSA (Infectious Diseases Society of America) guidance [6].

However, some of these options are suboptimal due to significant toxicity concerns (e.g., nephrotoxicity with colistin), unfavorable pharmacokinetic (PK) profiles, and increasing reports of resistance [7,8], while others have very limited clinical data. The recent IDSA guidelines underscore the precarious state of CRAB treatment, recommending combination therapies while explicitly noting the scarcity of high-quality evidence and the critical need for novel antimicrobial agents [9].

Eravacycline is a fully synthetic fluorocycline antibiotic approved for the treatment of complicated intra-abdominal infections. Its chemical structure confers stability against common tetracycline resistance mechanisms, such as efflux pumps and ribosomal protection, resulting in potent in vitro activity against a broad spectrum of MDR bacteria, including CRAB [10,11]. Studies have shown that eravacycline minimum inhibitory concentrations (MICs) against CRAB are generally 2- to 8-fold lower than those of tigecycline [12,13]. The pivotal IGNITE trials demonstrated non-inferiority of eravacycline to carbapenems for cIAI overall; however, these studies were not designed with *A. baumannii* as a primary target, and *A. baumannii* isolates were uncommon in the trial populations [14].

While its clinical efficacy for the approved indication is well established, real-world experience specifically addressing eravacycline use against CRAB is still limited. Recent literature, including case series and cohort studies, has begun to illustrate its potential as a salvage therapy for CRAB ventilator-associated pneumonia and bacteremia [15,16]. However, detailed reports focusing on its role in deep-seated abdominal infections, where effective source control is paramount, remain limited.

We report two cases of severe intra-abdominal infections caused by DTR CRAB that were successfully treated with eravacycline after the failure of multiple prior antimicrobial regimens. Our experience aims to contribute valuable clinical data to the evolving understanding of eravacycline’s role in managing these formidable infections.

## 2. Case Presentation

### 2.1. Case 1

#### 2.1.1. Past Medical History

A 75-year-old Caucasian man, independent in activities of daily living, had a medical history notable for chronic ischemic heart disease with permanent atrial flutter, for which he had undergone percutaneous coronary intervention with stent implantation 15 years earlier. Approximately 10 years before the current presentation, he had required mitral valvuloplasty for severe mitral regurgitation secondary to partial rupture of the posterolateral papillary muscle. His comorbidities also included chronic kidney disease, chronic obstructive pulmonary disease with an emphysematous phenotype, and a history of amiodarone-induced hyperthyroidism. He was a former smoker.

#### 2.1.2. Admission to Emergency Unit and First Hospitalization

In December 2024, the patient presented to the emergency department with new-onset jaundice and intermittent right upper quadrant abdominal pain. On admission, he was alert, fully oriented, afebrile, and breathing comfortably in room air. Physical examination revealed tenderness on deep palpation of the right upper quadrant, without peritoneal signs.

Laboratory tests showed a pattern consistent with obstructive jaundice: white blood cell (WBC) count 10,480/mm^3^ (65% neutrophils), hemoglobin 14.1 g/dL, platelets 264,000/mm^3^, serum creatinine 1.82 mg/dL, aspartate aminotransferase (AST) 122 U/L, alanine aminotransferase (ALT) 242 U/L, alkaline phosphatase 316 U/L, gamma-glutamyl transferase 657 U/L, amylase 143 U/L, lipase 58 U/L, C-reactive protein (CRP) 12.4 mg/dL, and total bilirubin 6.87 mg/dL (direct 5.39 mg/dL, indirect 1.48 mg/dL).

Abdominal ultrasound revealed dilation of the intrahepatic bile ducts in the left hepatic segments. A non-contrast-enhanced abdominal computed tomography (CT) scan showed an overdistended gallbladder with probable biliary sludge, dilation of the cystic duct, and mild dilatation of the intrahepatic bile ducts. The patient was admitted to the Department of Gastroenterology for further evaluation.

**Figure 1:**
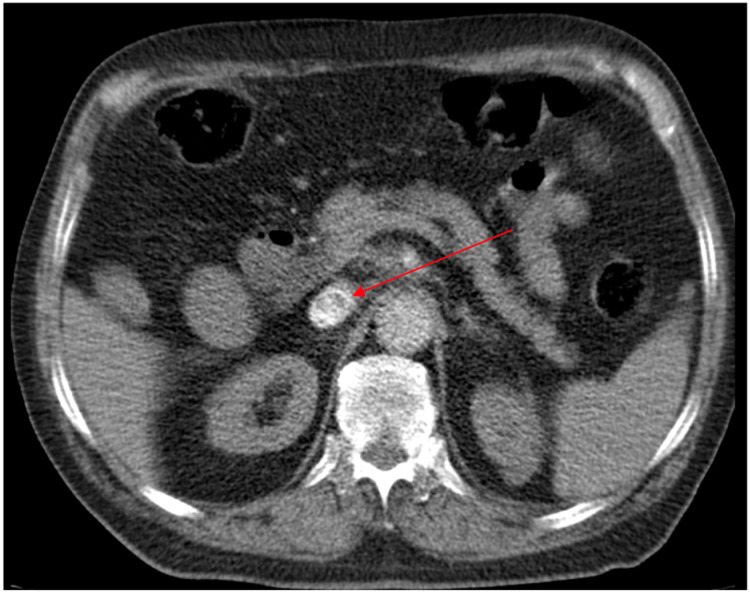
Contrast-enhanced abdominal CT demonstrating a concentrically thickened segment of the common bile duct with contrast enhancement at the pre- and intrapancreatic portion, extending longitudinally for about 1.7 cm (red arrow).

A contrast-enhanced abdominal CT revealed a concentrically thickened, contrast-enhancing segment of the common bile duct at the pre- and intrapancreatic portion, extending longitudinally for approximately 1.7 cm, causing near-complete luminal stenosis (Figure 1). These findings were highly suggestive of a distal cholangiocarcinoma. An endoscopic retrograde cholangiopancreatography (ERCP) was performed with placement of a metal stent for biliary drainage. The patient was discharged in improved clinical condition.

#### 2.1.3. Second Hospitalization and Bile Peritonitis

**Figure 2:**
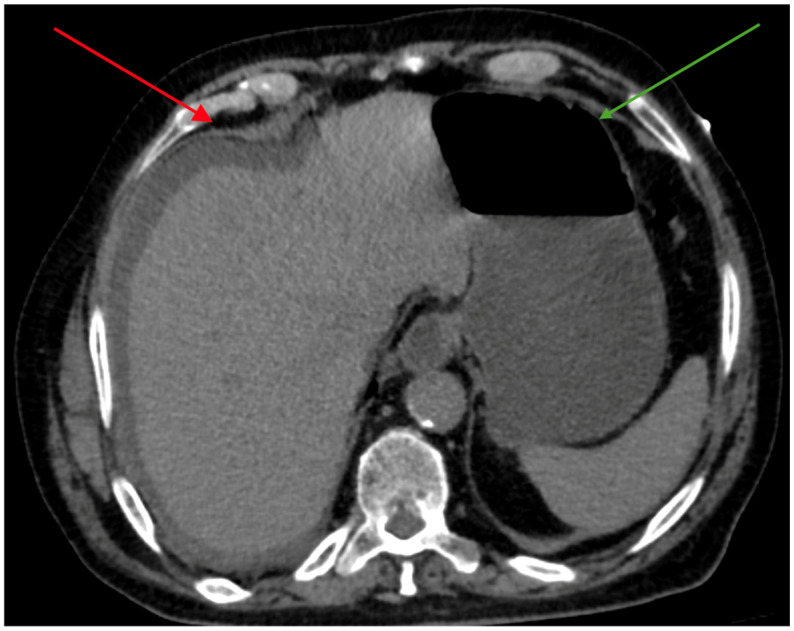
Contrast-enhanced abdominal CT demonstrating a perihepatic fluid collection (red arrow), consistent with bile peritonitis. Coexisting fluid–gas distension of the stomach (green arrow).

The patient was readmitted shortly thereafter for repeat tissue sampling of the biliary stricture. He underwent endoscopic ultrasound (EUS) with fine-needle biopsy (FNB) of the common bile duct. In the hours following the procedure, he developed worsening abdominal pain and hypotension (blood pressure 85/45 mmHg). Laboratory tests now showed WBC 8880/mm^3^ (90% neutrophils), serum creatinine 2.85 mg/dL, CRP 225 mg/L, procalcitonin (PCT) 5.15 µg/L, and lactate 4.1 mmol/L. Contrast-enhanced CT revealed a perihepatic fluid collection consistent with bile peritonitis, along with fluid–gas distension of the stomach (Figure 2).

Empirical antibiotic therapy with piperacillin/tazobactam (4.5 g every 8 h intravenous (IV)) was initiated, and the patient underwent urgent laparotomy. Intraoperatively, a pinpoint discontinuity of the common bile duct with bile leakage into the peritoneal cavity was identified. A T-tube was placed for external biliary drainage, and additional drains were inserted. Intraoperative cultures grew *Enterococcus faecalis*, *Escherichia coli*, and *Pseudomonas aeruginosa*. Based on these findings, antibiotic therapy was escalated to meropenem (1 g IV every 8 h) plus ampicillin (2 g IV every 4 h).

#### 2.1.4. Isolation of *Acinetobacter baumannii* and Eravacycline Therapy

After an initial phase of clinical improvement, the patient again deteriorated with increasing abdominal drainage and rising inflammatory markers. Bile cultures at this stage yielded CRAB (Table 1) and ampicillin-susceptible *Enterococcus faecium*. The isolation of CRAB from bile in the setting of postoperative biliary peritonitis, septic shock, and purulent intra-abdominal collections was interpreted as true infection rather than colonization. Antimicrobial therapy was modified to high-dose ampicillin/sulbactam (9 g every 8 h IV, administered as a 4 h infusion) to provide activity against both CRAB and *Enterococcus faecium*, in combination with meropenem (1 g every 8 h IV) to maintain broader Gram-negative coverage. High-dose sulbactam was selected to maximize pharmacodynamic exposure against CRAB, in line with current evidence and international recommendations.

Despite seven days of this regimen, inflammation remained poorly controlled, with CRP persisting at 172 mg/L and purulent output from the drains. Follow-up CT showed an increase in the perihepatic fluid collection, suggesting ongoing uncontrolled biliary leakage or superinfection. Meropenem was therefore discontinued, and targeted anti-CRAB therapy was intensified with the addition of eravacycline administered at a weight-based dose of 1 mg/kg (50 mg) intravenously every 12 h. This dosing regimen, calculated for a body weight of 50 kg, is consistent with the approved dosing for complicated intra-abdominal infections and supported by published pharmacokinetic data. Eravacycline was administered in combination with high-dose ampicillin/sulbactam. No dose adjustment was required based on renal or hepatic function. After the introduction of eravacycline, the patient showed progressive clinical improvement, with a decrease in C-reactive protein to 44.6 mg/L, resolution of hemodynamic instability, and removal of the T-tube, leading to resolution of septic shock. Clinical response was defined by defervescence, resolution of shock, and a marked reduction in inflammatory biomarkers. These improvements were sustained throughout the entire period of eravacycline therapy. The temporal association between eravacycline initiation and rapid clinical and laboratory improvement further supported the role of CRAB as the causative pathogen. Source control was attempted prior to CRAB isolation through surgical exploration and biliary drainage; however, a persistent enteric leak with residual infected collections, documented on contrast-enhanced CT, led to ongoing infection and necessitated prolonged antimicrobial therapy. As the leak was not amenable to further surgical correction, antimicrobial treatment was continued as a bridging strategy to maintain clinical stability in the setting of incomplete source control.

**Figure 3:**
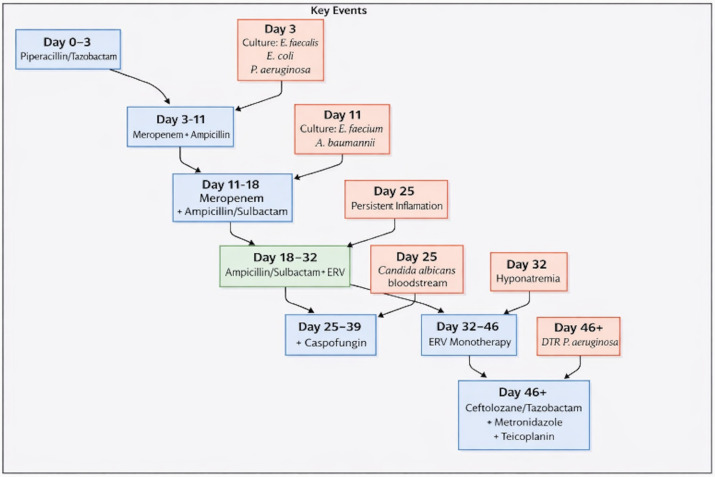
Antimicrobial regimens administered and microbiological isolates detected in cultures starting from the day of surgery (Day 0). Abbreviations: ERV, eravacycline; DTR, difficult-to-treat.

During this period, the patient developed candidemia, which was treated with caspofungin (70 mg IV loading dose on day 1, followed by 50 mg IV once daily). Due to the onset of hyponatremia, ampicillin/sulbactam was discontinued on postoperative day 32, while eravacycline was continued as monotherapy until day 46. A subsequent bile culture grew difficult-to-treat, resistant *Pseudomonas aeruginosa*, prompting discontinuation of eravacycline after a total of 28 days of therapy. The late postoperative course was marked by progressive metabolic acidosis, respiratory failure, and refractory shock, ultimately leading to death after 93 days of hospitalization despite microbiological control of the CRAB infection. Microbiological response was defined as clearance of CRAB from follow-up cultures. A detailed timeline of antimicrobial therapy and microbiological findings is provided in Figure 3.

### 2.2. Case 2

#### 2.2.1. Past Medical History and Disease Course

A 64-year-old Caucasian man had a medical history of lumbar discectomy and tonsillectomy. He was an active smoker and reported consuming approximately two alcohol units per day. Over the preceding five months, he experienced marked unintentional weight loss of 26 kg accompanied by anorexia. He was admitted to hospital with severe epigastric pain; laboratory tests revealed elevated amylase and lipase levels, and contrast-enhanced CT confirmed acute necrotizing hemorrhagic pancreatitis. Two months later, he developed fever, nausea, and vomiting. Repeat abdominal CT demonstrated a large peripancreatic fluid collection with intralesional air bubbles and fistulous communications, consistent with walled-off necrosis (WON).

#### 2.2.2. Admission and Clinical Deterioration

**Figure 4:**
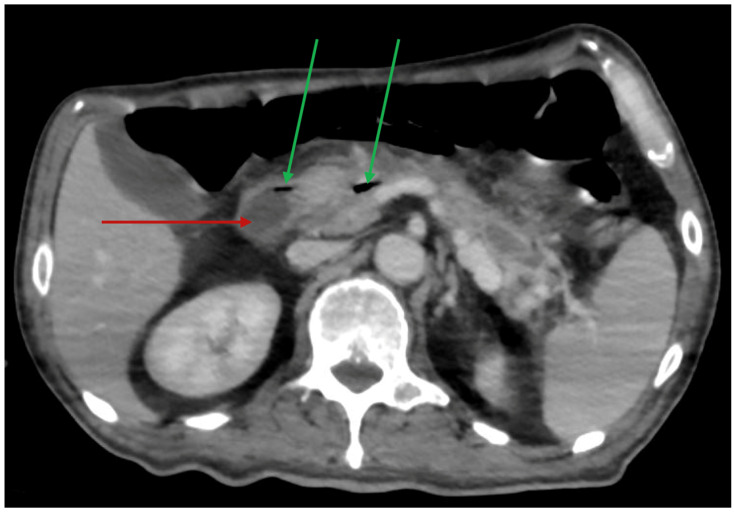
Contrast-enhanced abdominal CT performed on the first day of admission to the Gastroenterology Unit, showing a heterogeneous fluid-density collection in the pancreatic body–tail region (red arrow) with small intralesional air bubbles (green arrows), consistent with infected walled-off necrosis.

In June 2025, the patient was admitted to our Gastroenterology Unit for endoscopic management of the WON. On admission, he was alert but markedly asthenic and dehydrated. Laboratory tests showed a WBC count of 6440/mm^3^ (77% neutrophils), platelet count of 124,000/mm^3^, CRP 125 mg/dL, and PCT 0.926 µg/L. A contrast-enhanced CT scan confirmed a heterogeneous pancreatic collection with gas foci, consistent with infected necrosis (Figure 4). Empirical antimicrobial therapy with ceftriaxone (2 g every 24 h IV) and metronidazole (500 mg every 8 h IV) was initiated.

On hospital day 4, he developed acute respiratory failure and hypotension. Chest CT revealed bilateral pulmonary infiltrates with cavitations. He was intubated and transferred to the intensive care unit (ICU), where transthoracic echocardiography showed a severely reduced left ventricular ejection fraction (LVEF) of 20%. Bronchoalveolar lavage cultures yielded methicillin-susceptible *Staphylococcus aureus* (MSSA). Antimicrobial therapy was therefore switched to cefazolin (2 g IV every 8 h) in combination with meropenem (1 g IV every 8 h).

#### 2.2.3. Isolation of *Acinetobacter baumannii* and Eravacycline Therapy

After clinical stabilization in the ICU, the patient was transferred back to the Gastroenterology Unit. Shortly thereafter, he again developed fever, prompting repeat blood cultures that grew CRAB: vancomycin-resistant *Enterococcus faecium* (VRE), *Staphylococcus epidermidis*, and *Candida albicans*. Antimicrobial therapy was escalated to cefiderocol (2 g every 8 h IV, administered over 3 h), ampicillin/sulbactam (9 g every 8 h IV, administered over 4 h), teicoplanin (12 mg/kg every 12 h IV for three loading doses, followed by 12 mg/kg IV once daily) and caspofungin (70 mg loading dose, then 50 mg once daily IV). Given the presence of septic shock, persistent fever, and bacteremia in a postoperative patient with infected pancreatic necrosis, the isolation of CRAB was interpreted as true invasive infection rather than colonization.

Because of persistent fever and inflammatory markers despite this regimen, cefiderocol was replaced with colistin on day 27. Colistin was administered as a loading dose of 300 mg of colistin base activity (CBA), corresponding to 9 million international units (MIU) of colistimethate sodium, followed by a maintenance regimen of 150 mg CBA (4.5 MIU) twice daily, starting 12 h after the loading dose.

Percutaneous drainage of the pancreatic collection was subsequently performed. Culture of the drained material yielded CRAB and ampicillin-susceptible *Escherichia coli*. Despite drainage and combination antimicrobial therapy, the patient continued to have fever and elevated inflammatory markers. On day 32, colistin was discontinued, and eravacycline was initiated at 1 mg/kg (50 mg) IV every 12 h, based on the patient’s body weight of 50 kg, in combination with ampicillin/sulbactam as targeted salvage therapy against CRAB. No dose adjustment was required for renal or hepatic function.

The combination of eravacycline and effective percutaneous drainage resulted in rapid clinical and laboratory improvement, with defervescence and normalization of inflammatory biomarkers. Clinical response was defined by resolution of fever, normalization of inflammatory markers, and radiological reduction of the infected collection. Follow-up CT demonstrated a marked reduction in the size of the pancreatic collection. On day 45, all antibiotics were discontinued: ampicillin/sulbactam after 29 days, teicoplanin after 29 days, and eravacycline after 14 days of therapy. Microbiological response was defined as clearance of CRAB from follow-up cultures. The coordinated timing of source control and eravacycline initiation was considered essential for achieving infection resolution. The infection was considered resolved. The patient was discharged shortly thereafter in fair general condition, with improving nutritional status and exercise tolerance. At the first outpatient follow-up visit one month later, he remained clinically stable and afebrile, with normalized inflammatory markers and no clinical or radiological evidence of recurrence during subsequent follow-up.

## 3. Discussion

The management of severe IAIs caused by DTR CRAB represents a significant clinical challenge in contemporary practice. Our two cases provide compelling real-world evidence supporting the role of eravacycline as an effective salvage therapy in this context.

In both patients, eravacycline administration was associated with a decisive clinical turnaround. This was evidenced by the resolution of septic shock, defervescence, and a marked decline in serum inflammatory markers (CRP and PCT), all occurring after the failure of complex, broad-spectrum regimens that included carbapenems, ampicillin/sulbactam, and, in one case, cefiderocol and colistin. Although eravacycline susceptibility testing was not available in our laboratory and no in vitro MICs were obtained, its selection as salvage therapy was guided by published data showing potent activity against CRAB, with MICs typically lower than those of tigecycline, and by its approval and pharmacokinetic profile for complicated intra-abdominal infections. This aligns with the robust in vitro activity of eravacycline against CRAB, as documented in the literature [12,13]. In this context, eravacycline represented the only additional agent with a reasonable expectation of activity beyond colistin, and the close temporal relationship between its initiation and clinical improvement strongly supports a relevant therapeutic contribution.

In our series, CRAB isolates exhibited a classic multidrug-resistant phenotype, remaining susceptible only to colistin among the agents tested, thereby underscoring the paucity of viable alternatives. The decision to introduce eravacycline as a last-resort therapeutic option, despite the absence of local susceptibility data, reflects the urgent need for effective treatment options in critically ill patients with DTR CRAB IAIs and is consistent with emerging clinical evidence supporting its use in other severe CRAB infections.

Our findings are consistent with the growing body of clinical reports on eravacycline for CRAB. In particular, the recent case series by Mimram et al. described three critically ill patients with DTR *A. baumannii* ventilator-associated pneumonia who were successfully managed with eravacycline, providing an important clinical signal in a setting with very limited options [15]. Bronchopulmonary pharmacokinetic studies in healthy volunteers have shown that eravacycline achieves higher concentrations in epithelial lining fluid than in plasma, indicating favorable distribution into the lower respiratory tract and supporting its potential role in pneumonia [17]. This pharmacological profile is consistent with a multicenter real-world study conducted in respiratory departments, in which eravacycline demonstrated high clinical success and microbiological eradication, particularly in infections caused by MDR *A. baumannii* and *Klebsiella pneumoniae*, with a very low incidence of adverse events [18]. In parallel, a narrative review on new antibiotics for Gram-negative pneumonia by Bassetti et al. highlighted eravacycline, alongside cefiderocol and plazomicin, as a promising option for the management of severe respiratory infections caused by *A. baumannii*, further reinforcing its potential utility in this difficult-to-treat context [19]. Furthermore, a large retrospective study by Chen et al. reported favorable outcomes in lung transplant recipients with CRAB infections treated with eravacycline, underscoring its potential utility even in profoundly immunocompromised hosts [16].

In addition to these pneumonia-focused data, a retrospective case series by Buckley et al. reported the use of eravacycline in ten patients with diverse *A. baumannii* infection types, offering further real-world evidence that this agent can be employed across a spectrum of clinically relevant syndromes and helping to frame our intra-abdominal cases within a broader clinical experience [20].

A key issue in both patients was the difficulty in achieving timely and definitive source control. In both patients, antibiotic courses with ampicillin/sulbactam and eravacycline were considerably longer than those recommended for uncomplicated IAIs, reflecting the complexity of the clinical scenario rather than a deliberate deviation from guideline-based practice. In Case 1, persistent collections and the suspicion of ongoing occult leakage, together with severe comorbidities and surgical risk, limited the options for more aggressive or repeated source control procedures and prompted the continuation of broad-spectrum therapy as a “bridging” strategy to maintain clinical stability.

In Case 2, the presence of a large, initially poorly drainable pancreatic WON and the patient’s critical condition also contributed to a prolonged treatment course. Eravacycline and high-dose ampicillin/sulbactam were maintained until effective percutaneous drainage could be achieved and sustained radiological improvement was documented, at which point all antibiotics were stopped without relapse. From a stewardship perspective, the extended duration of therapy in our cases should be viewed as a weakness of the management pathway and underlines the importance of timely and optimal source control, which remains the cornerstone of cIAI treatment. In Case 1, despite the eventual identification of a persistent enteric fistula, the combination of advanced age, significant cardiovascular and respiratory comorbidities, and the complex biliary–enteric anatomy markedly limited the feasibility of repeated or more aggressive surgical or radiological interventions. In Case 2, the presence of an extensive, initially poorly drainable pancreatic WON, together with severe hemodynamic instability and respiratory failure, contributed to extended treatment durations.

Importantly, eravacycline was well tolerated in both patients despite prolonged exposure (28 and 14 days, respectively), without clinically relevant hepatic, renal, or gastrointestinal toxicity. This favorable safety profile may represent a practical advantage over other last-line options for DTR CRAB, particularly in critically ill patients in whom both the margin for toxicity and the possibility of definitive source control are limited.

This favorable tolerability profile is a significant advantage over other last-line agents, such as colistin, and is consistent with the safety data reported in clinical trials and real-world studies [14,21].

Our cases illustrate the potential of eravacycline in the intra-abdominal compartment, a site for which it is officially approved, even when CRAB is the causative pathogen. In our series, eravacycline use was associated with complete clinical recovery and sustained microbiological cure in Case 2, whereas in Case 1, microbiological eradication was achieved, but the patient ultimately died due to progressive multiorgan failure and the overall severity and complexity of his underlying condition, reflecting the high baseline risk that often characterizes patients with severe *A. baumannii* infections.

A limitation of our report is its inherent nature as a case series, which prevents definitive conclusions about efficacy compared to other regimens. Furthermore, in complex, polymicrobial infections, it can be difficult to isolate the specific contribution of one antimicrobial agent to the overall clinical outcome. However, the clear microbiological and clinical response coinciding with eravacycline initiation in both cases provides a strong argument for its therapeutic role.

## 4. Conclusions

The management of severe IAIs caused by DTR *A. baumannii* remains among the most difficult challenges in contemporary infectious diseases. In our two critically ill patients with complex, polymicrobial IAIs due to DTR CRAB, clinical improvement followed the introduction of eravacycline after failure of multiple conventional and salvage regimens. In Case 2, eravacycline combined with effective percutaneous drainage resulted in complete recovery without relapse, whereas in Case 1, microbiological eradication was achieved, but the patient ultimately died due to progressive multiorgan failure and the severity of underlying comorbidities.

Eravacycline, a fully synthetic fluorocycline with potent in vitro activity against CRAB and approved for complicated IAIs, emerges from these cases as a rational and relatively safe salvage option when standard therapies have been exhausted. The absence of relevant adverse events despite prolonged exposure further supports its favorable tolerability profile.

At the same time, these cases highlight that potent antimicrobial therapy cannot compensate for suboptimal source control. Prolonged antibiotic courses in our patients were largely driven by frailty, anatomical complexity, and the limited feasibility of surgical or radiological interventions and should be regarded as a weakness of the management pathway. This reinforces the central role of timely and effective source control, multidisciplinary decision-making, and careful antimicrobial stewardship in the management of complicated IAIs.

Finally, although based on only two cases and therefore not definitive, our observations provide a plausible rationale for considering eravacycline as a valuable component of salvage therapy in critically ill patients with DTR CRAB intra-abdominal infections, always in conjunction with the best achievable source control.

## Figures and Tables

**Table 1:** Susceptibility profile of *Acinetobacter baumannii* isolated from bile in Case 1.

Antibiotic	MIC	Sensitivity
Amikacin	>32 µg/mL	R
Ciprofloxacin	>1 µg/mL	R
Colistin	≤1 µg/mL	S
Gentamicin	>4 µg/mL	R
Imipenem	>8 µg/mL	R
Levofloxacin	>8 µg/mL	R
Meropenem	>8 µg/mL	R
Tobramycin	>8 µg/mL	R
Trimethoprim/Sulfamethoxazole	>8/152 µg/mL	R

## Data Availability

No new data were created or analyzed in this study. Data sharing is not applicable to this article.

## Abbreviations

The following abbreviations are used in this manuscript:

ALT	alanine aminotransferase
AST	aspartate aminotransferase
CBA	colistin base activity
cIAI/cIAIs	complicated intra-abdominal infection(s)
CRAB	carbapenem-resistant Acinetobacter baumannii
CRP	C-reactive protein
CT	computed tomography
DTR	difficult-to-treat resistant
ELF	epithelial lining fluid
ERCP	endoscopic retrograde cholangiopancreatography
ERV	eravacycline
ESBL	extended-spectrum β-lactamase
EUS	endoscopic ultrasound
FNB	fine-needle biopsy
HAP	hospital-acquired pneumonia
IAIs	intra-abdominal infections
ICU	intensive care unit
IDSA	Infectious Diseases Society of America
IV	intravenous
LVEF	left ventricular ejection fraction
MBL	metallo-β-lactamase
MDR	multidrug-resistant
MIC	minimum inhibitory concentration
MIU	million international units
MSSA	methicillin-susceptible Staphylococcus aureus
OXA	class D carbapenem-hydrolyzing oxacillinase β-lactamase
PCT	procalcitonin
PK	pharmacokinetics/pharmacokinetic
VAP	ventilator-associated pneumonia
VRE	vancomycin-resistant Enterococcus
WBC	white blood cell (count)
WON	walled-off necrosis
WHO	World Health Organization
